# Triptolide Causes Spermatogenic Disorders by Inducing Apoptosis in the Mitochondrial Pathway of Mouse Testicular Spermatocytes

**DOI:** 10.3390/toxics12120896

**Published:** 2024-12-10

**Authors:** Jiantao Zhao, Maosheng Cao, Haisheng Yi, Guitian He, Tong Chen, Lingyun Liu, Kaimin Guo, Yin Cao, Chunjin Li, Xu Zhou, Boqi Zhang, Hongliang Wang

**Affiliations:** 1 Department of Andrology, The First Hospital of Jilin University, Jilin University, Changchun 130021, China; 17862891160@163.com (J.Z.); yihs21@mails.jlu.edu.cn (H.Y.); tmxlly@jlu.edu.cn (L.L.); gkm7631703@jlu.edu.cn (K.G.); caoyin21@jlu.edu.cn (Y.C.); 2 College of Animal Sciences, Jilin University, Changchun 130062, China; caoms21@mails.jlu.edu.cn (M.C.); guitianhe0812@163.com (G.H.); ctokay@163.com (T.C.); llcjj158@163.com (C.L.); xzhou65@vip.sina.com (X.Z.)

**Keywords:** triptolide, testicular injury, spermatogenesis impairment, spermatocytes, mitochondria

## Abstract

Triptolide (TP) is a diterpenoid compound extracted from the traditional Chinese medicinal herb Tripterygium wilfordii. It has antitumor and anti-inflammatory effects and stimulates immunity. However, its serious side effects, especially reproductive toxicity, limit its clinical application. This study employed a testicular injury model established by intraperitoneally injecting TP (0.2 mg/kg) in C57BL/6J male mice (age = 7–8 weeks) for 14 days. The control and TP mice’s testicular tissues were subjected to transcriptome sequencing to assess potential testicular damage mechanisms. Based on the transcriptome sequencing results and relevant literature reports, further experiments were performed. In addition, to alleviate triptolide-induced testicular damage, we treated the mice with N-acetyl-L-cysteine (NAC). The acquired data revealed that compared with the control mice, the TP-treated mice’s testes indicated severe damage. Transcriptome sequencing identified differentially expressed genes that showed enrichment in cell differentiation, apoptotic process, cell cycle, glutathione (GSH) metabolism, and the p53 signaling pathway. Furthermore, TUNEL assays and Western blot analysis showed that in the TP mice’s testicular tissues, the spermatocytes had mitochondrial pathway apoptosis as well as abnormal mitochondrial morphology and structure. Triptolide induces oxidative stress in testicular tissue by enhancing pro-oxidative systems and inhibiting antioxidant systems. NAC reduced testicular damage and apoptosis by alleviating TP-induced oxidative stress. This study also employed a GC2 cell line for in-vitro analyses, and the results were consistent with the in vivo experiments. This study provides evidence for alleviating TP’s adverse effects on the male reproductive system for better clinical application.

## 1. Introduction

In recent years, traditional Chinese medicine and its extracts have garnered increasing attention for their therapeutic effects in the fields of autoimmune diseases and tumors. TP is a diterpenoid from the medicinal plant *Tripterygium wilfordii* and has anti-inflammatory, immunosuppressive, and antitumor properties. Recently, it has been observed that treating rheumatoid arthritis in mice with TP nano-composite hydrogel can reduce leukocyte infiltration, alleviate cartilage destruction, and decrease the concentrations of the inflammatory cytokines interleukin (IL)-1β, tumor necrosis factor-α (TNF-α), and IL-6, thereby effectively improving joint inflammation [1]. Furthermore, TP can be targeted specifically to dendritic cells via dendritic cell-derived exosomes, which could induce immunosuppression as well as reduce inflammation and damage in ulcerative colitis and rheumatoid arthritis [2]. Moreover, Jing Cai et al. [3] found that TP induces pyroptosis in head and neck cancers by inhibiting mitochondrial hexokinase II expression, which promotes the translocation of Bcl-xL/Bcl-2-associated death promoter (Bad) and Bcl-2-associated-X-protein (Bax), and the activation of caspase-3, which then cleaves and activates gasdermin E (GSDME). Although TP has significant anti-inflammatory, immunosuppressive, and antitumor activities, it is highly toxic to organs such as the kidneys, liver, testes, and ovaries [4], and its reproductive toxicity is an especially serious concern.

The testes are male internal reproductive organs with the functions of spermatogenesis and steroid hormone synthesis. *Tripterygium wilfordii*-induced male reproductive toxicity is similar to that of Bisphenol A [5], as it reduces the body and testicular weight in rats and disrupts the structure of seminiferous tubules. Additionally, it can decrease the testosterone conversion rate in a time- and dose-dependent manner and inhibit the expression of key enzymes involved in testosterone synthesis [6]. Most importantly, it significantly reduces sperm count [7] and motility, leading to reversible male infertility [8]. Triptolide, the active component of *Tripterygium wilfordii*, exhibits similar toxicity. It has been observed that TP reduces organ indices of the testes, epididymis, seminal vesicles, and preputial glands [9,10,11,12] in mice and promotes wrinkles on the testes’ surface with irregular thickening of the tunica albuginea [10]. Furthermore, microscopically, TP has been observed to reduce the diameter of seminiferous tubules, epithelium height, and lumen diameter [9,12,13], which leads to deformation, atrophy [10], disordered arrangement, and vacuolization of the seminiferous tubules [9]. In addition, TP enlarges the interstitial spaces between the seminiferous tubules [10], promotes collagen accumulation in the testicular interstitium [10], and reduces the numbers of various germ cells [9,10,11,13,14], such as spermatogonia cells, spermatogonial stem cells, meiotic spermatocytes, and Sertoli cells. This affects late spermatogenesis events [9], resulting in decreased sperm count and increased malformation rates [13]. Fertility studies have indicated that TP use is associated with a reduced number of offspring [9]. Furthermore, it lowers the levels of spermatogenesis-related hormones, including the luteinizing hormone [13] and serum testosterone [14]. Moreover, TP also damages the blood–testis barrier [11]. Clinical reports have indicated that the long-term, high-dose application of Tripterygium glycoside tablets results in azoospermia. Therefore, exploring the mechanisms of TP-induced reproductive toxicity and identifying strategies to mitigate its side effects are significant. Mechanistically, triptolide induces reproductive system toxicity through various mechanisms, including oxidative stress [12], apoptosis [12], endocrine disruption [6], mitochondrial damage [15], and DNA damage [16].

Apoptosis is a genetically controlled, autonomous, and orderly cell death [17] that maintains homeostasis. There are three classic apoptosis pathways, including the endoplasmic reticulum, mitochondrial, and death receptor pathways. Among them, the mitochondrial pathway is the most common pathway for apoptosis. The mitochondrial pathway is regulated by proteins of the Bcl-2 family [18], including the pro-apoptotic Bak, Bad, Bid, Bax, and Bim, and the anti-apoptotic Bcl-x, Bcl-2, Bcl-w, and Bcl-xL. Under normal conditions, Bcl-xL and Bcl-2 form heterodimers with Bak and Bax, thereby inhibiting mitochondrial apoptosis [19]; however, once stimulated, cytoplasmic Bax undergoes mitochondrial translocation where it promotes pore formation in the mitochondrial membrane, reducing membrane potential and promoting cytochrome c (Cytc) release [12]. The decrease in membrane potential initiates apoptosis, while the released Cytc binds apoptotic protease activating factor-1 (Apaf-1) to form an ATP-dependent apoptosome. Procaspase-9, in its zymogen state, is recruited to the apoptosome and undergoes autocatalytic activation to caspase-9, leading to the activation of downstream caspase-3, which cleaves substrates such as α-tubulin, actin, PARP, and lamin, thereby causing apoptosis. TP-induced apoptosis has been studied in cancer cells [20,21], macrophages [22], and synovial cells [23]. Furthermore, some studies have also evaluated that TP induces apoptosis in testicular cells; however, these studies were primarily focused on Leydig cells [24,25,26,27,28], Sertoli cells [11,12,14,29,30], and the blood–testis barrier, and a few were focused on germ cells and in vivo experiments. Therefore, this investigation is based on animal experiments and aims to elucidate the underlying mechanism of how TP causes spermatogenic dysfunction by damaging germ cells, providing support for the improved clinical application of TP.

## 2. Material and Methods

### 2.1. Animals and Treatment

C57BL/6J mice (male, weight = 20–25 g, age = 7–8 weeks) were procured from the Experimental Animal Center of Jilin University and maintained in the pathogen-free animal laboratory of Jilin University under controlled conditions of 22 ± 2 °C temperature, 60 ± 5% humidity, and a 12:12 h light:dark cycle with free food and water access. The animal experiment protocols were reviewed and authorized by the Animal Experiment Ethics Committee of Jilin University [SY202409019]. All the mice were randomly categorized into the control and the TP groups (n = 6, respectively). Referring to the previous report [9], the TP mice were intraperitoneally injected with 0.2 mg/kg of TP (GIpbio, GC14402) daily for 14 days in succession, while the controls received equal volumes of saline with 1% DMSO. All mice were euthanized with sodium pentobarbital after 24 h of the final administration. The testes and epididymis samples were collected and photographed.

Furthermore, to elucidate the protective effect of N-acetyl-L-cysteine (NAC; Yuanye, Shanghai, China) on TP-induced testicular damage, the mice were also randomly categorized into the following 4 groups (n = 6/group): control, TP, TP + NAC, and NAC groups. Their breeding environment and drinking and eating protocol were the same as before. The NAC and TP + NAC groups were given intraperitoneal injections of NAC (100 mg/kg/d) for 28 days, after which all mice were euthanized with sodium pentobarbital, and the testes and epididymis samples were harvested and photographed.

### 2.2. Organ Index

The body weights and the weights of the testes and epididymis of the mice were measured using a balance. Furthermore, the testis and epididymis indices were measured. The following formula was employed to evaluate the organ index: organ weight/body weight × 100%.

### 2.3. Histological and Immunohistochemical Staining

The mouse testes were preserved in 4% paraformaldehyde, dehydrated using gradient methanol, embedded in wax, sectioned, and stained with hematoxylin and eosin (H&E) to identify the pathological alterations in the testicular tissue under a light microscope. Moreover, the diameters of the seminiferous tubules, epithelial heights, and tunica albuginea thicknesses were also measured. For immunohistochemistry (IHC), the tissue sections were treated overnight with primary antibodies (anti-B lymphoma Mo-MLV insertion region 1 [Bmi1; MCE, 1:200] and anti-synaptonemal complex protein 3 [Sycp3; Bioss, 1:100]) at 4 °C, treated with secondary antibodies at ambient temperature for 30 min, probed with horseradish peroxidase-labeled streptavidin for 30 min at ambient temperature, and then reacted with a DAB chromogenic solution for color development.

### 2.4. Epididymal Sperm Count

The epididymis was transferred into an Eppendorf tube (1.5 mL) containing phosphate-buffered saline (PBS; 1 mL) and finely minced with ophthalmic scissors to fully disperse the sperms and form a suspension. Then, 10 µL of this suspension was placed on a hemocytometer to count the number of sperm using a light microscope.

### 2.5. Transcriptomic Analysis

The total testicular tissue RNA was isolated with the help of TRIzol (Thermo Fisher, Fremont, CA, USA), and the mRNA was purified via Dynabeads Oligo (dT). Then, the mRNA was reverse-transcribed into cDNA via Superscript II reverse transcriptase (Invitrogen, cat. Fremont, CA 1896649, USA). The Illumina NovaseqTM 6000 platform (LC-BioTechnology Co., Ltd., Hangzhou, China) was then employed for paired-end sequencing (PE150). Furthermore, the R packages DESeq2 and edgeR were used for the identification of differentially expressed genes (DEGs) using the criteria of an absolute *p*-value < 0.05 or fold change ≥ 2. Gene Ontology (GO), and KEGG enrichment analyses of the DEGs were performed.

### 2.6. TUNEL Assay

Apoptosis was assessed via TUNEL assays. The testicular paraffin sections were incubated with a proteinase K working solution at an ambient temperature for 20 min, washed for 5 min in PBS thrice, treated with a membrane permeabilization solution (20 µL 10% sodium citrate + 20 µL 10% Triton-X100 + 1980 µL ddH_2_O) for 10 min at an ambient temperature, and washed again thrice with PBS for 5 min. Based on the number of slides and the tissue size, Reagents 1 and 2 from the TUNEL kit were mixed at a ratio of 1:9 and applied to the slides to cover the tissue. For the negative control, only 50 µL of Reagent 2 was added. The sections were incubated for 60 min at 37 °C in a water bath, rinsed thrice with PBS in the dark for 5 min, and then mounted using an anti-fade mounting medium comprising DAPI (Beyotime Biotechnology, Nantong, China). The slides were visualized and imaged under an Olympus inverted fluorescence microscope. The ImageJ software 2023 was employed for the quantitative analysis of images.

### 2.7. Cell Culture and Flow Cytometry

The GC2 spd (Procell Life Science & Technology Co., Ltd., Wuhan, China) cell line was cultured with DMEM/F12 (Sigma-Aldrich, St. Louis, MO, USA) and supplemented with 10% FBS (ABW, Uruguay) and 1% Penn/Strep (Proteintech, Wuhan, China). The Annexin V-FITC Apoptosis Detection Kit (Beyotime Biotechnology, Nantong, China) was employed as per the kit’s guide to assess apoptosis. Briefly, the GC2 cells were grown in 6-well plates and exposed to TP (100 nmol/L) for 24 h. The cells were rinsed with PBS, trypsinized, centrifuged for 5 min at 1000 g to collect the pellet, and resuspended in Annexin V-FITC binding buffer (195 μL), followed by treatment with propidium iodide (PI; 10 μL) and Annexin V-FITC (5 μL) at an ambient temperature away from light for 10–20 min before analysis via a flow cytometer. A mitochondrial membrane potential (MMP) Detection Kit (JC-1) (Beyotime Biotechnology, Nantong, China) was employed per the kit’s guide to assess the mitochondrial membrane potential (MMP) levels. After different treatments, GC2 cells were incubated with a JC-1 fluorescence probe followed by the analysis of flow cytometry.

### 2.8. Malondialdehyde (MDA) and Glutathione (GSH) Assay

The tissue and cellular GSH and MDA levels were measured using the GSH and GSSG Assay Kit and Lipid Peroxidation (MDA) Assay Kit (Beyotime Biotechnology). Briefly, testis tissues from the control and TP mice were collected, and MDA and GSH levels were determined as per the kit’s guide. For the in vitro analysis, briefly, GC2 cells were propagated in 6-well plates and treated accordingly for 24 h. Then, the cells were washed with PBS, trypsinized, and centrifuged for 5 min at 1000× *g* to collect the pellet. The cellular GSH and MDA levels were determined as per the kit’s guide. The protein concentration was normalized using the Omni-EasyTM Ready-to-Use BCA Protein Assay Kit (Shanghai Epizyme Biomedical Technology Co., Ltd., Shanghai, China).

### 2.9. Intracellular Reactive Oxygen Species Detection

Intracellular levels of reactive oxygen species (ROS) were assessed using a kit (Beyotime Biotechnology). Briefly, in a 6-well plate, the GC2 cells were propagated and then treated accordingly for 24 h. Following aspiration of the media, DCFH-DA (1 mL diluted to 1:1000 in a serum-free media) was added per well at 37 °C for 20 min. After three washes with a serum-free medium, the cells were treated with 200 μL of DAPI (Beyotime Biotechnology). The green fluorescence was visualized under a fluorescence microscope (Olympus, IX71, Tokyo, Japan) and quantified via ImageJ.

### 2.10. Quantitative Real-Time PCR

The whole testicular tissue RNA was harvested with the help of the TRIzol reagent (Takara Biotechnology, Beijing, China), whereas for the cellular RNA extraction, buffers A (38.2 g Guanidine hydrochloride + 0.213 g Morpholine ethanesulfonic acid (MES) + 0.372 g Ethylenediaminetetraacetic acid (EDTA)), B (96% ethanol), C (3 mmol/L Sodium acetate), and D (70% ethanol) were employed. The RNA was evaluated spectrophotometrically at 260 and 280 nm and reverse-transcribed to cDNA using SuperScript II reverse transcriptase (Invitrogen, cat. 1896649, USA), and then amplified using SYBR Green. The relative gene expression was assessed via the 2^−ΔΔCT^ method using PPIA as an endogenous reference. The sequences of gene primers (Comate Bioscience Co., Ltd., Changchun, China) employed are as follows: Mus-PPIA F: GAAGCCATGGAGCGTTTTGG R: ATTGCGAGCAGATGGGGTAG; Mus-Sycp3 F: AAAGATGGTGCCTGGTGGAA R: CAGCAACATCTTCTTCTGAACCA; Mus-Gpx4: F: CCGTCTGAGCCGCTTACTTA R: GGCTGAGAATTCGTGCATGG; Mus-Txndc2 F: TGTGGATTTCTCAGCCGCTT R: TCCTCAGTGTCCACCTCCAA; Mus-Homx2 F: TCGGAGGGGGTAGATGAGTC R: TTCTGCTCGGTCATGTGCTT; Mus-Aptx F: CTTACCGTGGGCCTCCATTT R: TGCTATCACCTTCTCCCCCA; Mus-Lcn2 F: ACAACCAGTTCGCCATGGTAT R: AAGCGGGTGAAACGTTCCTT; Mus-Romo1 F: GTCTCAGGATCGGAATGCGG R: CCAATGGCCATGAAAGTGCC; Mus-Cyp11a1 F: CTAAAGGACTTTCCCTGCGCT R: AGAGGTACCAGCTCCCTTTC; Mus-Ho-1 F: GAACCCAGTCTATGCCCCAC R: GGCGTGCAAGGGATGATTTC; Mus-Jun F: TGGGCACATCACCACTACAC R: TCTGGCTATGCAGTTCAGCC

### 2.11. Western Blot

The proteins from the testicular tissue and GC2 cells were isolated with the RIPA lysis buffer (Solarbio, Beijing, China), denatured by heating for 5 min at 95 °C, isolated via SDS-PAGE, and transferred to a 0.45% PVDF membrane, which was then blocked using a quick-blocking solution (Epizyme, Shanghai, China) at an ambient temperature for 15 min, and then treated overnight with primary antibodies (rabbit anti-Sycp3 (Bioss, bs-10660R, 1:1000), rabbit anti-Bax (Cell Signaling, Cat. 2772T, 1:1000), rabbit anti-Bcl-2 (MCE, HY-P80029, 1:1000), rabbit anti-Cleaved-caspase3 (Cell Signaling, Cat. 9661T, 1:1000), and mouse anti-β-actin (Bioworld, AP0060, 1:1000) at 4 °C. Then, the membranes were rinsed with Tris-Buffered Saline Tween (TBST) 4 times, each time for 6 min, probed at an ambient temperature with the corresponding horseradish peroxidase-conjugated secondary antibodies (Bioworld, 1:8000 dilution) for 1 h, washed thrice with TBST, each time for 6 min, and reacted with an enhanced chemiluminescence solution (Epizyme, Shanghai, China) for 2 min. The Tanon-5200 automatic digital gel imaging system (Tanon Science & Technology Co., Ltd., Shanghai, China) was employed for visualizing the protein bands, which were quantitatively analyzed using ImageJ.

### 2.12. Cell Viability Assay

CCK-8 assays (Beyotime Biotechnology) were utilized to assess cell viability. Briefly, the GC2 cells were propagated in a 96-well plate and then treated with TP or NAC. Then, the CCK-8 reagent (10 µL) was added to each well in the dark for 1 h before the optical density was read using an Eon microplate reader at 450 nm to assess cell viability.

### 2.13. Ultrastructural Observation

The control and TP mice’s testes were cut into small rectangular pieces (1 × 3 × 0.5 mm) and transferred into EP tubes (2 mL) containing 2.5% glutaraldehyde fixative solution for 12 h at 4 °C. Furthermore, the cultured and treated cells were directly scraped off and transferred to EP tubes (1.5 mL), centrifuged at a low speed (1000–3000 rpm) to form a pellet, and mixed with 2.5% glutaraldehyde fixative solution at 4 °C for 12 h. The mitochondrial morphology and structure of the testicular tissue and GC2 cells from different treatment groups were observed under an electron microscope.

### 2.14. Molecular Docking

To investigate the interaction modes and binding energy of TP with Gpx4 and Nrf2, the Autodock Vina 1.2.2 (http://autodock.scripps.edu/, accessed on 2 March 2024) docking software was employed. TP’s molecular structure was acquired from the PubChem database (https://pubchem.ncbi.nlm.nih.gov/, accessed on 2 March 2024), whereas the structures of Gpx4 (PDB ID: 2GS3; resolution: 1.90 Å) and Nrf2 (PDB ID: 7O7B) were downloaded from the Protein Data Bank (http://www.rcsb.org/, accessed on 2 March 2024). All ligand and protein files were saved in the PDBQT format, with the addition of polar hydrogens and the removal of water molecules. Then, the grid box was centered such that it covered each protein’s domain and accommodated the molecule’s free movement. The interface pocket was then set to a cubic pocket with 30 × 30 × 30 Å dimensions and 0.05 nm of grid spacing.

### 2.15. Statistical Measurements

All the statistical assessments were conducted via SPSS 26 (IBM, Armonk, NY, USA), and the data are indicated as the mean ± standard deviation of three replicates. For inter-group difference comparison, an independent sample t-test was conducted, while for multiple-group comparison, one-way ANOVA was performed. *p* < 0.05 depicted statistical differences; *p* < 0.01 depicted statistically significant differences.

## 3. Results

### 3.1. TP Caused Testicular Damage and Spermatogenesis Impairment in Mice

**Figure 1:**
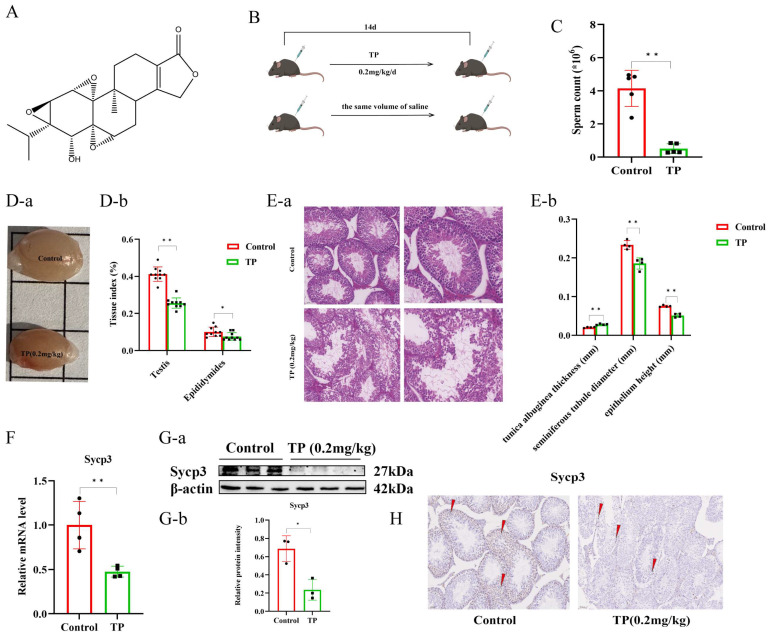
Triptolide-induced testicular damage and spermatogenic disorders. (**A**) The chemical structure of triptolide. (**B**) A flowchart of medication administration. (**C**) The epididymal sperm count (n = 5). (**D**) The size of the testis (**a**) and organ index (**b**). (**E**) H&E staining, the seminiferous tubule diameter, the epithelium height (**a**), and the tunica albuginea thickness of the testes (**b**). (**F**) The relative mRNA level of Sycp3 (n = 4). (**G**) The Western blot of Sycp3 (n = 3) (**a**,**b**). (**H**) The immunohistochemistry staining of testes. * *p* < 0.05, ** *p* < 0.01 vs. control (means ± SEM).

TP is a diterpenoid from the medicinal plant *Tripterygium wilfordii,* and its chemical structural formula is shown in Figure 1A. After 14 consecutive days of intraperitoneal TP (0.2 mg/kg) injections (Figure 1B), there was significant damage to the testicles. The epididymal sperm count analysis indicated that the TP group’s sperm count was markedly reduced compared to that of the control mice (Figure 1C). The mice’s testicular volume and the testis and epididymis indices were significantly decreased (Figure 1D). Furthermore, H&E staining also indicated a reduced number of spermatogenic epithelial cells, disorganized cell arrangement, decreased seminiferous tubule diameter and epithelial height, and increased tunica albuginea thickness (Figure 1E).

Moreover, qRT-PCR analysis showed that relative to the controls, the mRNA levels of Sycp3 (meiotic spermatocytes marker) were significantly reduced in the TP group (Figure 1F). The Western blot analysis was consistent with the qRT-PCR results, which revealed that the protein levels of Sycp3 were downregulated in the TP group (Figure 1G). In addition, IHC analysis indicated that, compared with the control mice, TP mice had few Sycp3-positive cells (Figure 1H). Altogether, these data suggest that TP causes testicular morphological abnormalities and structural disorders, and also reduces the number of spermatocytes and epididymal sperm.

### 3.2. Transcriptome Sequencing

**Figure 2:**
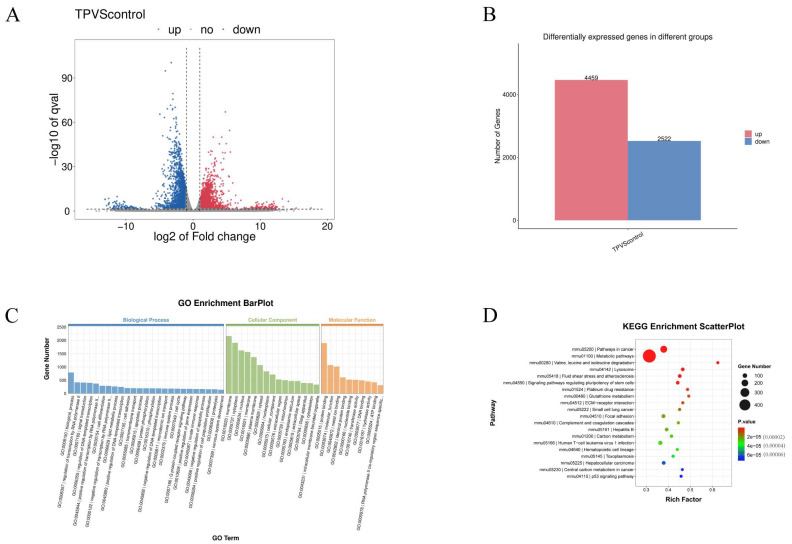
Transcriptomic analysis of testes. (**A**) A volcano plot of differential expression genes (DEGs). (**B**) The number of DEGs. (**C**) GO enhancement analyses of the DEGs. (**D**) KEGG enhancement analyses of the DEGs.

To investigate the mechanisms by which TP causes testicular damage and spermatogenesis impairment in mice, transcriptomic sequencing was performed on the testes of the control and the TP groups (3 samples/group). The data revealed that relative to the control group, the TP-treated testicular tissue had 4459 upregulated and 2522 downregulated genes, with statistically significant differences (*p* < 0.05 and the absolute value of log2-fold change >1) (Figure 2A,B). Moreover, the GO and KEGG enrichment analyses found that the DEGs were primarily enriched in pathways linked to cell differentiation, the cell cycle, GSH metabolism, apoptosis, and the p53 signaling pathway (Figure 2C,D).

### 3.3. TP Induces Apoptosis in Testicular Spermatocytes and GC2 Cells via the Mitochondrial Pathway

**Figure 3:**
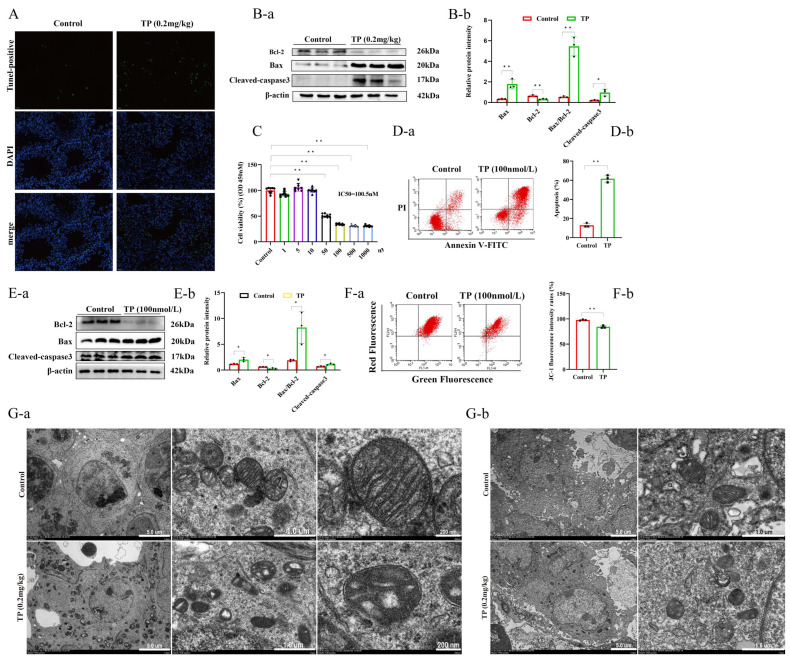
Triptolide caused apoptosis of the mitochondrial pathway in testicular spermatogonia and GC2 cell lines. (**A**) TUNEL staining of testes. (**B**) Western blot analysis of Bax, Bcl-2, cleaved-caspase3 (n = 3) (**a**,**b**). (**C**) The cell viability of GC2 cells treated with different concentrations of triptolide. (**D**) The detection of apoptosis in GC2 cells by flow cytometry (**a**,**b**). (**E**) Western blot analysis of Bax, Bcl-2 (**a**), and cleaved-caspase3 (n = 3) (**b**). (**F**) Flow cytometric analysis was used to examine the mitochondrial membrane potential (MMP) levels in GC2 cells (**a**,**b**). (**G**) The electron microscopic observation of testicular (**a**) and GC2 cell mitochondria (**b**). * *p* < 0.05, ** *p* < 0.01 vs. control (means ± SEM).

Based on transcriptomic sequencing results combined with previous related reports [12], it was hypothesized that cell apoptosis reduces the number of spermatocytes. This hypothesis was verified via Tunnel assay on testicular tissue. The results showed that TP-treated mice’s testicular tissue had a large number of positive (apoptotic) cells, whereas the control group had almost no positive cells (Figure 3A). Furthermore, these positive cells indicated morphological characteristics of spermatocytes. Since the mitochondrial pathway represents the most common form of apoptosis, this research study confirmed whether TP induced spermatocyte apoptosis via the mitochondrial pathway by assessing the expression of Bcl-2, Bax, and cleaved-caspase3 proteins as well as the Bax to Bcl-2 ratio using Western blot analysis. The data showed that the Bax expression and Bax/Bcl-2 ratio were notably upregulated, while the Bcl-2 levels were substantially downregulated, and the cleaved-caspase3 protein level was elevated (Figure 3B). In addition, a mouse testicular spermatocyte cell line (GC2) was also employed to verify these results at the cellular level. First, the impact of TP on cell viability was confirmed, which markedly and dose-dependently inhibited cell viability, with an IC_50_ of 100.5 nmol/L at 24 h (Figure 3C). Combined with the morphological changes of cells under the microscope, 100 nmol/L of TP was selected for further analyses. GC2 cells were treated with 100 nmol/L TP for 24 h, stained with Annexin V-FITC/PI, and then assessed via flow cytometry to detect apoptosis levels. The data revealed that the number of apoptotic cells markedly enhanced after 24 h of 100 nmol/L TP treatment, with the proportion reaching 60% (Figure 3D). The protein levels of Bax, Bcl-2, and cleaved-caspase3, as well as the Bax/Bcl-2 ratio in both groups, were further measured using a Western blot analysis. Quantitative analysis showed consistent results with the in vivo analysis (Figure 3E). We further detected mitochondrial membrane potential (MMP) in GC2 cells after TP treatment because the decrease in mitochondrial membrane potential is necessary for cell apoptosis. MMP levels in GC2 cells were significantly reduced after TP treatment (Figure 3F). This study also assessed the mitochondrial morphology and structure of the testicular tissue and GC2 cells treated with TP under an electron microscope, which revealed that, relative to the controls, the TP-treated group exhibited an abnormal mitochondrial structure, including mitochondrial atrophy and cristae disorganization (Figure 3G). Altogether, these results indicate that TP induces mitochondrial pathway apoptosis in testicular spermatocytes and GC2 cells.

### 3.4. TP Induced Oxidative Stress in Mouse Testicular Tissue and the GC2 Cell Line by Enhancing the Pro-Oxidant System and Inhibiting the Antioxidant System

**Figure 4:**
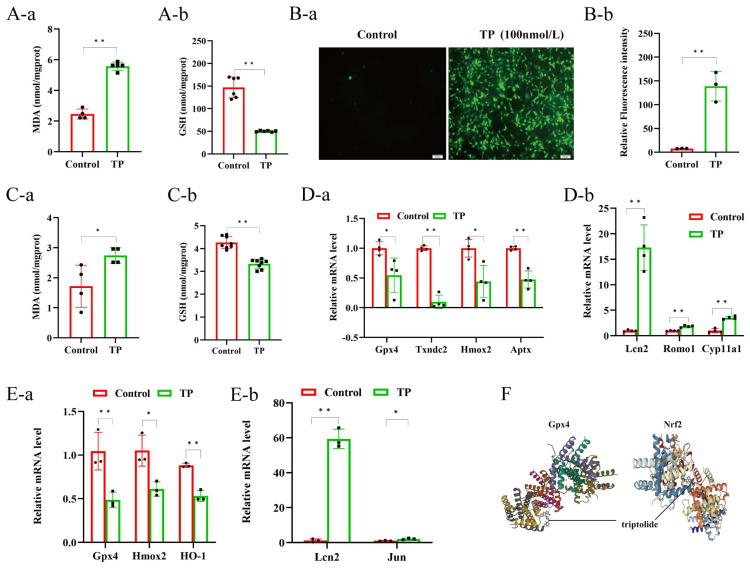
Triptolide led to oxidative stress in mouse testicular tissue and GC2 cell lines by enhancing the oxidative system and attenuating the antioxidant system (**A**) The MDA (**a**) and GSH (**b**) levels of testicular tissue. (**B**) The intracellular ROS levels of GC2 cell lines (**a**,**b**). (**C**) The MDA and GSH levels of GC2 cell lines (**a**,**b**). (**D**) The relative mRNA level of Gpx4, Txnd2, Hmox2, Aptx, Lcn2, Romo1 (**a**), and Cyp11a1 in testicular tissue (n = 4) (**b**). (**E**) The relative mRNA level of Gpx4, Hmox2, HO-1, Lcn2 (**a**), and Jun in GC2 cell lines (n = 3) (**b**). (**F**) The molecular docking of TP, Gpx4, Nrf2. * *p* < 0.05, ** *p* < 0.01 vs. control (means ± SEM).

The literature has indicated that oxidative stress is crucially associated with cell apoptosis. To determine whether the action of TP involves oxidative stress, MDA and GSH levels in the testicular tissues of the two groups were measured. This showed that, relative to the controls, the MDA levels in the testicular tissues of the TP mice were significantly elevated, whereas the GSH levels were significantly reduced (Figure 4A). In the in vitro analysis, the GC2 cells were incubated with TP (100 nmol/L) for 24 h and then probed with DCFH-DA at 37 °C for 20 min. The intensity of green fluorescence, indicating intracellular ROS levels, was evaluated using fluorescence microscopy. The acquired data revealed highly intense green fluorescence in the TP group relative to the controls, with a significant difference (Figure 4B). The same method was used to determine the MDA and GSH levels in GC2 cells with different treatments, and the results were consistent with those in the testicular tissues (Figure 4C). Moreover, to verify that TP damages the antioxidant system and activates the pro-oxidant system, qRT-PCR was used to examine the levels of relevant pro-oxidant and antioxidant genes. The data revealed that in the TP-treated testicular tissues, the mRNA levels of the antioxidant factors glutathione peroxidase 4 (Gpx4), thioredoxin domain-containing 2 (Txndc2), heme oxygenase 2 (Hmox2), and aprataxin (Aptx) were downregulated, while those of the pro-oxidant genes lipocalin 2 (Lcn2), reactive oxygen species modulator 1 (Romo1), and cytochrome P450 family 11 subfamily A member 1 (Cyp11a1) were markedly upregulated (Figure 4D). Moreover, in the in vitro analysis, the TP-treated cells revealed the reduced expression of antioxidant genes heme oxygenase 1 (HO-1), Gpx4, Hmox2, and the enhanced expression of pro-oxidant genes Lcn2 and Jun Proto-Oncogene (Jun) (Figure 4E). In addition, molecular docking found that TP has strong binding affinities with Gpx4 (−8.994 kcal/mol) and Nrf2 (−7.758 kcal/mol) (Figure 4F). Overall, these data revealed that TP promoted oxidative stress in vivo and in vitro by enhancing the pro-oxidant system and inhibiting the antioxidant system.

### 3.5. TP Induced Spermatogenic Dysfunction in Mice by Activating Oxidative Stress-Mediated Mitochondrial Apoptotic Pathways

**Figure 5:**
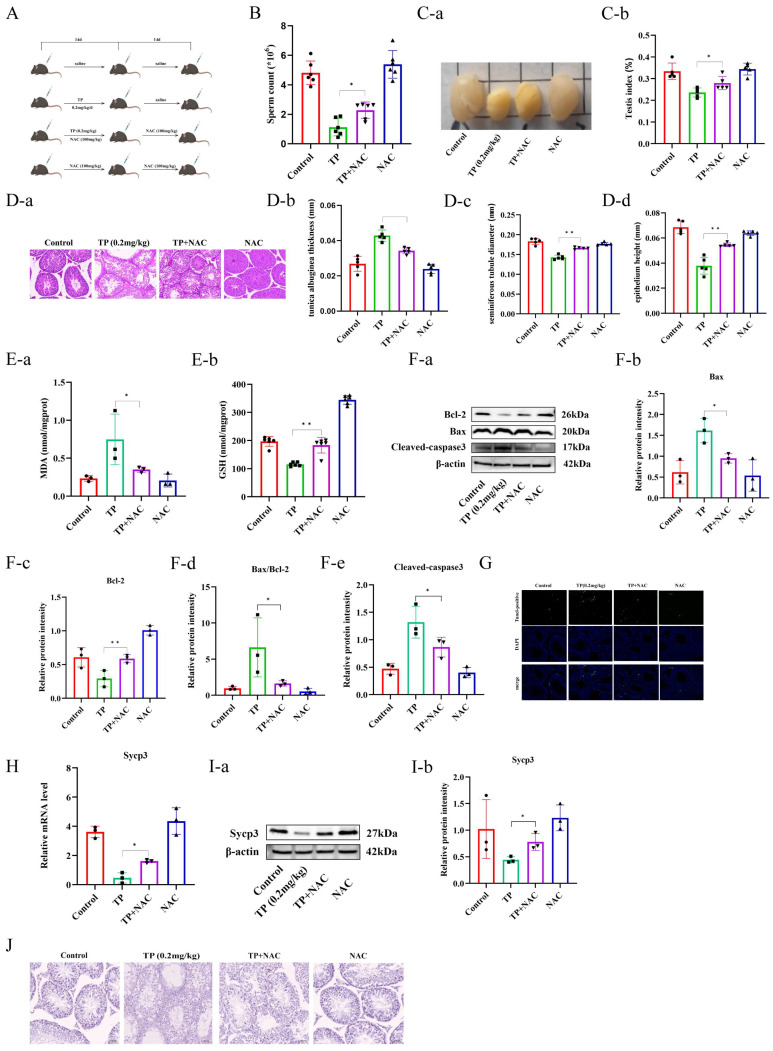
The activation of oxidative stress-mediated apoptosis in the mitochondrial pathway by triptolide caused impaired spermatogenesis in mice. (**A**) A flowchart of medication administration. (**B**) The epididymal sperm count (n = 6). (**C**) The size of the testis (**a**) and the testis index (**b**). (**D**) H&E staining (**a**), the seminiferous tubule diameter (**b**), the epithelium height (**c**), and the tunica albuginea thickness of testes (**d**). (**E**) The MDA (**a**) and GSH (**b**) levels of testicular tissue. (**F**) Western blot analysis of Bax, Bcl-2, cleaved-caspase3 of testicular tissues with different treatments (n = 3) (**a**–**e**). (**G**) TUNEL staining of the testes. (**H**) Relative mRNA levels of Sycp3 (n = 3). (**I**) Western blot analysis of Sycp3 of testicular tissues with different treatments (n = 3) (**a**,**b**). (**J**) Immunohistochemistry staining of the testes. * *p* < 0.05, ** *p* < 0.01 vs. control (means ± SEM).

**Figure 6:**
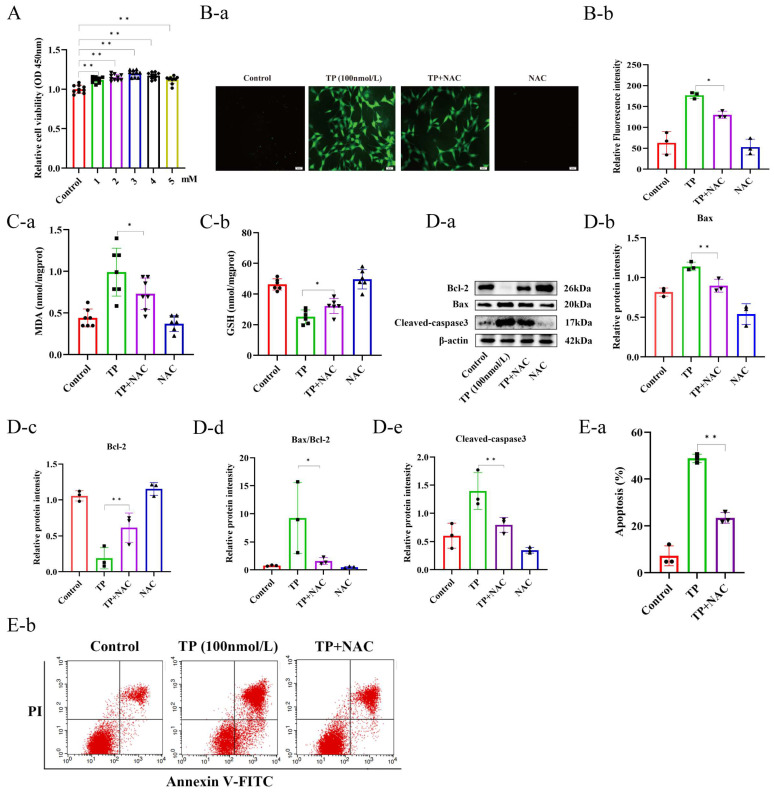
The inhibition of oxidative stress alleviates triptolide-induced apoptosis of GC2 cell lines. (**A**) The cell viability of GC2 cells treated with different concentrations of NAC (1–5 mM). (**B**) The intracellular ROS levels of GC2 cell lines (**a**,**b**). (**C**) The MDA (**a**) and GSH (**b**) levels of GC2 cell lines. (**D**) Western blot analysis of Bax, Bcl-2, cleaved-caspase3 of GC2 cells with different treatments (n = 3) (**a**–**e**). (**E**) The detection of apoptosis in GC2 cells by flow cytometry (**a**,**b**). * *p* < 0.05, ** *p* < 0.01 vs. control (means ± SEM).

To investigate whether TP induces spermatogenic dysfunction in mice by promoting the apoptosis of spermatocytes in the testes, an antioxidant therapy was performed on the mice using the intraperitoneal injection of NAC (Figure 5A). Sperm counts in the epididymis indicated that sperm abundance in the TP + NAC mice was greater than in the TP mice (Figure 5B). It was observed that compared to the TP mice, the TP + NAC mice had partially restored testicular volume and an increased testicular index (Figure 5C). Furthermore, the histological analysis of testicular tissues via H&E staining indicated that NAC mitigated the damage caused by TP, as evidenced by an increased number of spermatogenic cells, more organized arrangement, as well as significantly improved seminiferous tubule diameter, epithelial height, and diminished tunica albuginea thickness (Figure 5D). The MDA and GSH levels were measured in mice’s testicular tissues from different groups, which indicated that the TP + NAC group had reduced MDA levels compared to the TP group, and GSH levels were markedly restored, indicating that NAC successfully alleviated oxidative stress in testicular tissues (Figure 5E). Moreover, whether antioxidant therapy mitigated testicular cell apoptosis was examined by Western blotting analysis of Bcl-2, Bax, and cleaved-caspase-3 protein levels, as well as the Bax/Bcl-2 ratio. It was observed that, relative to the TP group, the TP + NAC group had reduced levels of cleaved-caspase-3 and Bax proteins, enhanced Bcl-2 protein levels, and a decreased Bax/Bcl-2 ratio (Figure 5F). Furthermore, the TUNEL assays of testicular tissues revealed a notable reduction in positive cells (apoptotic cells) in the TP + NAC mice compared to the TP mice (Figure 5G). Moreover, qRT-PCR and Western blotting data suggested that the TP + NAC mice had increased Sycp3 mRNA and protein levels compared with the TP mice (Figure 5H,I). IHC data further found that the TP + NAC mice had an increased number of spermatocytes in the seminiferous tubules than the TP mice (Figure 5J). This study also screened the appropriate in vitro concentration of NAC by treating the GC2 cells with various NAC concentrations for 24 h, followed by the measurement of cell viability with CCK-8 assays. This revealed that cell viability was increased with all NAC concentrations, with the highest cell viability observed at 3 mmol/L (Figure 6A), which was selected for subsequent experiments. To verify whether NAC mitigates TP-induced oxidative stress, the cells were co-treated with 3 mmol/L of NAC and TP for 24 h. After treatment, we assessed oxidative stress-related indicators, including ROS, MDA, and GSH levels. The data revealed that, in comparison with the TP group, the fluorescence intensity in the TP + NAC group was reduced (Figure 6B), MDA levels were decreased, and GSH levels were increased (Figure 6C). Next, whether cell apoptosis was alleviated was also assessed. Western blotting determined that, compared to the TP-treated cells, the TP + NAC treated cells had reduced Bax and Cleaved-caspase-3 levels, enhanced Bcl-2 expression, and a reduced Bax/Bcl-2 ratio (Figure 6D). Furthermore, flow cytometry was employed to assess the levels of apoptosis in cells from different treatment groups. The results showed a reduction in apoptotic cells and a significantly lower apoptosis rate in the TP + NAC group relative to the TP group (Figure 6E). Altogether, TP was observed to induce spermatogenic dysfunction by promoting the mitochondrial apoptotic pathway in spermatogonia and GC2 cells by increasing oxidative stress.

## 4. Discussion

This study employed animal models and transcriptome sequencing, which revealed that TP induces apoptosis in testicular spermatocytes, which decreases the number of spermatocytes, thereby reducing sperm count. Per our knowledge, this investigation is the first to indicate how TP damages reproductive cells in animals via in vivo and in vitro analyses.

*Tripterygium wilfordii* is an ancient herbal medicine that has been used in Asia for centuries. TP is a major active component of *Tripterygium wilfordii*, which was first extracted and isolated in 1972 [31]. Several investigations have observed TP toxicity in the testicles through the damaging of steroid hormone synthesis and the blood–testis barrier, as well as spermatogenesis by affecting Leydig, Sertoli, and germ cells. This investigation found that 14 days of intraperitoneal TP (0.2 mg/kg administration) markedly reduced the testicular volume and testis index in mice, which is consistent with previous reports. Furthermore, IHC staining, qRT-PCR, and Western blot analyses of testicular samples indicated a reduced number of spermatocytes. To verify this result, transcriptome sequencing was carried out. The DEGs were enriched in pathways such as cell apoptosis, cell cycle, GSH metabolism, differentiation, and p53 signaling. Furthermore, the TUNEL assay confirmed apoptosis in spermatocytes.

Mitochondria are the metabolic centers within cells, providing energy for various life activities. Furthermore, mitochondria are also associated with various cell death pathways, including ferroptosis [32,33,34,35], necroptosis [36,37,38], pyroptosis [39,40,41], and apoptosis [12]. The pro-apoptotic protein Bax has been observed to stimulate apoptosis by enhancing the permeability of the mitochondrial membrane and secreting apoptotic factors into the cytoplasm, including cytochrome c. The change of mitochondrial outer membrane potential and cytochrome c release from mitochondria is necessary, which are the events required for mitochondrial apoptosis. In contrast, the anti-apoptotic Bcl-2 provides protection by dimerizing with Bax to inhibit its activity. The equilibrium between Bax and Bcl-2 determines cell survival or apoptosis. Therefore, the modulation of Bcl-2 family proteins has become a hot topic in apoptosis and drug development research. Shashank Dadsena and others [42] discovered that polyunsaturated lipid materials are enriched near the Bax/Bak apoptotic pores, and by regulating Bax/Bak pore-forming activity, they promoted the permeabilization of the mitochondrial outer membrane. In terms of drug development, for nearly two decades, innovative drug research targeting the Bcl-2 protein family has focused on anti-apoptotic proteins, particularly the globally innovative Bcl-2 selective inhibitor Venetoclax [43]. Here, it was observed that the expression of the Bax protein in the testicular tissue of TP-treated mice was raised, while that of Bcl-2 was reduced, thereby increasing the Bax/Bcl-2 ratio and reducing mitochondrial membrane potential, suggesting that TP induces apoptosis via mitochondrial modulation. The in vitro results were consistent with the in vivo analyses. Yu Wang et al. [12] also found that TP promoted apoptosis in mouse testicular Sertoli cell lines via the mitochondrial pathway. Mitochondria typically appear oval or elongated and structurally comprise the outer and inner membranes and the outer and inner chambers, with inward projections of the inner membrane called “cristae”. Mitochondria are highly dynamic organelles that maintain structural balance through continuous division and fusion. The functions of mitochondria are closely related to their morphology. This investigation revealed that, relative to the control group, the mitochondrial morphology in the TP-treated testicular tissue and GC2 cells had varying degrees of shrinkage and cristae blurring, suggesting that TP affects the shape and structure of mitochondria. Zhiyan Qin et al. employed Mito-Tracker Red CMXRos to observe mitochondrial morphology in TM4 cells treated with TP and found that mitochondria transformed from tubular to fragmented forms. However, the present study did not assess alterations in mitochondrial function, which is a limitation of this research.

Helmut Sies [44] coined the term oxidative stress, which refers to the damage caused to biological systems due to an imbalance between antioxidant defenses and oxidant generation. Oxidative stress has been linked with a variety of diseases, such as radiation-induced lung injury [45], idiopathic pulmonary fibrosis [46], atherosclerosis [47,48], hypertension [49], paraquat poisoning [45], chronic obstructive pulmonary disease [50], and type 2 diabetes [51]. ROS include singlet oxygen, superoxide (O_2_^−^), hydrogen peroxide (H_2_O_2_), peroxynitrite (ONOO^−^), ozone, and hydroxyl radicals (·OH). ROS are the by-products of biological aerobic metabolism and are the executors of tissue damage caused by oxidative stress. ·OH and ONOO^−^ have been found to oxidize structural proteins, membrane lipids, nucleic acids, and enzymes, thereby causing cellular dysfunction and eventual death. Lipid peroxidation produces MDA, which indicates the oxidative stress level of the body. In comparison, GSH is an antioxidant within the body that significantly decreases with oxidative stress. Here, it was found that compared with the control mice, the TP-treated mice had markedly elevated MDA and decreased GSH levels in the testicular tissue, suggesting that TP promotes oxidative stress in testicular tissue, consistent with previous studies. Several studies have revealed that TP can dose-dependently enhance ROS and MDA in TM4 cells and decrease GSH levels. The present research study found that TP markedly enhanced ROS levels within GC2 cells, elevated MDA concentrations, and notably reduced GSH levels. The removal of lipid peroxides by GSH depends on Gpx4 [52], which is closely related to ferroptosis, necroptosis, pyroptosis, apoptosis, and PARP-1-dependent cell death. The in vivo analyses of the current study indicated that TP increased the levels of pro-oxidant genes such as Lcn2, Romo1, and Cyp11a1 but inhibited antioxidant genes including Gpx4, Txndc2, Hmox2, and Aptx. Moreover, these data were in line with the in vitro experiments, with TP upregulating pro-oxidant genes (Lcn2 and Jun) and inhibiting antioxidant genes (Gpx4, Hmox2, and HO-1). 

NAC is a clinical mucolytic drug that has been in use for more than 60 years. Furthermore, it also serves as a nutritional supplement to stabilize mood, prevent cancer, and alleviate Hashimoto’s thyroiditis. NAC is an acetylated form of L-cysteine and acts as an important antioxidant, predominantly by promoting GSH. After entering the cells, NAC is deacetylated to produce cysteine, which combines with glutamate and glycine to form GSH, thereby exerting antioxidant effects. The literature has reported that NAC can alleviate the TP-induced elevation of ROS and decrease cell viability. Here, the 28 days of NAC treatment markedly alleviated TP-induced testicular injury and spermatogenic disorders by improving the testicular volume and index, the microscopic structure, the spermatocyte count, and the epididymal sperm count. Therefore, although NAC is an old drug, it can be used to effectively mitigate testicular toxicity induced by TP.

## 5. Conclusions

In summary, this study revealed that exposure to TP leads to testicular atrophy in mice, with a significantly reduced diameter of seminiferous tubules, epithelial height, and tunica albuginea thickness. Furthermore, the number of spermatocytes, as well as the sperm count, were also reduced. Mechanistically, TP activates oxidative stress, inducing apoptosis via the mitochondrial pathway in spermatocytes, thereby reducing their numbers and impairing spermatogenesis. Moreover, TP causes abnormalities in mitochondrial morphology and structure. This study provides insights into alleviating testicular toxicity caused by TP.

Male infertility is exhibiting trends of rising incidence, declining semen quality, and the diversification of causes. Therefore, studying the mechanisms of male infertility and finding ways to improve it is of particular urgency. Recent studies have found that triptolide can also induce PANoptosis [22], pyroptosis [3], and ferroptosis [53], providing new insights into preventing and controlling the reproductive toxicity of triptolide. In addition, celastrol, a triterpenoid compound also extracted from Tripterygium wilfordii, has attracted increasing attention for its remarkable effects in improving metabolic syndromes [54], offering a wide range of prospects for innovative applications within the family of Tripterygium wilfordii preparations.

## Data Availability

All data generated or analyzed during this study are included in this article. Further inquiries can be directed to the corresponding author.
